# Criteria for early diagnosis of third molar agenesis: a retrospective radiographic study

**DOI:** 10.1590/2177-6709.28.3.e2321322.oar

**Published:** 2023-07-17

**Authors:** Margaux DUMAS, Pedro MARIANO-PEREIRA, Iman BUGAIGHIS, Paulo FERNANDES-RETTO, Luís PROENÇA

**Affiliations:** 1 Private practice (Bagnols sur Cèze, France).; 2 Egas Moniz School of Health and Science, Orthodontic Department (Caparica, Almada, Portugal); 3 Egas Moniz Center for Interdisciplinary Research (Caparica, Almada, Portugal); 4 The Libyan Authority for Scientific Research (Tripoli, Libya).; 5 Egas Moniz School of Health and Science, Quantitative Methods for Health Research Unit (Caparica, Almada, Portugal).

**Keywords:** Third molar agenesis, Diagnosis, Chronological age, Dental age, Skeletal age

## Abstract

**Objective:**

To explore the association between chronological, dental and skeletal ages and early diagnosis of third molars agenesis.

**Material and Methods:**

This retrospective radiographic study comprised a sample of 282 Portuguese patients (122 males and 160 females) who sought orthodontic treatment between 2007 and 2018. Each participant had panoramic and lateral cephalometric radiographs performed before and after the age of 14 years. The chronological age was categorized into three intervals between 11.0 and 13.11 years of age. The full eruption of the four-second molars was used as a criterion in determining dental age. Skeletal age was verified by the vertebral maturation method. The diagnosis of agenesis of third molars was initially performed by observation of the initial panoramic radiography undertaken before the age of 14 years. Subsequently, the diagnosis of agenesis of third molars was confirmed by visualizing the second panoramic radiography, obtained after the age of 14 years. The association between the accuracy of the diagnosis and the chronological, dental and skeletal ages was evaluated using the chi-square test, at a 5% significance level.

**Results:**

No significant association between chronological age and alterations in the diagnosis of third molar agenesis was identified. However, there was a significant association between third molar agenesis and both dental age (*p*<0.001) and skeletal age (*p*=0.006).

**Conclusion:**

The eruption of the four-second molars and the peak of growth could be considered as criteria for early diagnosis of third molar agenesis, whereas chronological age is not a reliable diagnostic indicator.

## RESUMO

**Objetivo:**

Explorar a associação entre as idades cronológica, dentária e esquelética e o diagnóstico precoce da agenesia dos terceiros molares.

**Material e Métodos:**

Este estudo radiográfico retrospectivo compreendeu uma amostra de 282 pacientes portugueses (122 homens e 160 mulheres) que procuraram tratamento ortodôntico entre 2007 e 2018. Cada participante tinha radiografias panorâmicas e cefalométricas laterais realizadas antes e depois dos 14 anos de idade. A idade cronológica foi categorizada em três intervalos entre 11,0 e 13,11 anos de idade. A erupção completa dos quatro segundos molares foi usada como critério para determinar a idade dentária. A idade esquelética foi verificada pelo método de maturação das vértebras cervicais. O diagnóstico de agenesia de terceiros molares foi inicialmente realizado pela observação da radiografia panorâmica inicial, realizada antes dos 14 anos de idade. Posteriormente, o diagnóstico de agenesia de terceiros molares foi confirmado pela visualização da segunda radiografia panorâmica, obtida após os 14 anos de idade. A associação entre a acurácia do diagnóstico e as idades cronológica, dentária e esquelética foi avaliada por meio do teste qui-quadrado, com nível de significância de 5%.

**Resultados:**

Não foi identificada associação significativa entre idade cronológica e alterações no diagnóstico de agenesia de terceiros molares. No entanto, houve associação significativa entre agenesia de terceiro molar e idade dentária (p<0,001) e idade óssea (p=0,006).

**Conclusão:**

A erupção dos quatro segundos molares e o pico de crescimento podem ser considerados critérios para o diagnóstico precoce da agenesia do terceiro molar, enquanto a idade cronológica não é um indicador diagnóstico confiável.

## INTRODUCTION

Selective tooth agenesis is one of the most prevalent developmental anomalies in humans. It can arise following disruptions at the early stages of tooth development, i.e. initiation and proliferation.^1^ Congenital absence of the third molar is the most prevalent type of hypodontia, with reported rates reaching 50% in some affected groups.^2^ In addition, Hellman^3^ reported that subjects with congenitally missing third molars are 13 times more prone to have agenesia of other teeth. Third molar germ formation usually starts around 11 years of age in about 90% of individuals, and it commonly emerges in the oral cavity at 18-20 years of age.^4^

It is generally accepted that agenesis of permanent teeth is strongly associated with the development of malocclusion.^5^ Therefore, clinicians, in particular orthodontists, ought to evaluate the whole dentition, including the existence or absence of a third molar.^6^ Moreover, in orthodontics, it has become increasingly recognized that beginning a treatment at the optimal time can be as critical as selecting a specific treatment protocol.^7^ ^,^ ^8^ Biological indicators such as sexual maturation, chronological age,^9^ dental development,^10^ and skeletal development,^11^ ^,^ ^12^ are some of the criteria most commonly used to identify the maturation stage, thereby contributing to the planning of an orthodontic treatment at the optimal time.^7^

Many researchers have attempted to identify the earliest age at which a third molar germ can be radiographically visualized.^13^ The age of onset of third molar development is very variable,^14^ and there is no consensus among authors about the chronological age at which a third molar tooth can be considered congenitally missing.^15^ ^,^ ^16^ ^,^ ^17^ The most frequently reported critical age is 14 years.^15^ ^,^ ^16^ However, Richardson^17^ observed that in the absence of a third molar germ at 10 years of age, the probability of the tooth being congenitally missing is 50%. Bolaños et al.^14^ proposed that the radiographic diagnosis of agenesia can be performed at 13 years of age. Nevertheless, a weak correlation between chronological age and development of the third molar has been observed.^14^ ^,^ ^18^

Estimating dental age in children and adolescents is based mainly on dental calcification observed in radiographs and the timing of dental eruption.^19^ Generally, panoramic radiographs are used to evaluate the stages of crown and root development.^10^ Published studies show that third molar congenital absence varies in different populations^14^ ^,^ ^20^ Several studies have reported a significant association between calcification delay and eruption of first premolar or second molar with third molar agenesis.^15^ ^,^ ^17^ ^,^ ^19^

Good quality lateral cephalometric radiographs, with a clear view of the cervical spine, have been widely used to evaluate the stage of Cervical Vertebral Maturation (CVM) and to correlate this with the stage of skeletal maturity. The advantage of this approach is that the same cephalometric radiograph is used for both purposes, which obviates the need for a second radiograph, thereby reducing the degree of radiation exposure. A significant amount of studies had verified that the CVM method is a reliable means of assessing circumpubertal growth levels and the phases of skeletal maturity. Baccetti et al.^11^ ^,^ ^12^ described a six-stage CVM assessment technique in which posteroanterior developing morphological alterations of the lower borders of the second, third and fourth cervical vertebrae were visualized throughout growth. CVM assessment has become the most widely used method for assessing skeletal age.^12^ ^,^ ^21^ ^,^ ^22^ However, there is a clear lack of studies that explore the role of skeletal age in diagnosing agenesis of the third molars. In this context, the main objective of the present study was to explore the association between chronological, dental and skeletal age and early diagnosis of third molar agenesis. The following null hypotheses were tested:

1. It is not possible to make a reliable diagnosis of third molar agenesis in patients aged between 11.0 and 13.11 years using chronological age alone.
2. Dental age, defined by the eruption of the four second molars, cannot be considered a diagnostic criterion of third molar agenesis.
3. Skeletal age, determined by the maturation of cervical vertebrae, cannot be used as a criterion in the diagnosis of third molar agenesis.

## MATERIAL AND METHODS

This retrospective observational study was approved by the Ethics Committee of Egas Moniz School of Health and Science (Reference number 680), and written consents were obtained from the included subjects prior to undertaking the investigation. 

The study cohort was selected using the pre-orthodontic records of 2,960 individuals seeking orthodontic treatment who attended the Egas Moniz Dental Clinic between 2007 and 2018. The inclusion criteria stipulated Portuguese individuals of both sexes who had good quality digital panoramic and lateral cephalometric radiographs at two time points: the first radiograph was taken between 11 and 13.9 years of age; the second panoramic radiograph was obtained after 14 years of age. None of the participants had undergone extraction of third molars during the evaluation period; none had cleft lip and palate, and none presented with a syndrome or craniofacial abnormalities affecting dental development. Any participants with poor quality radiographs or in which the anatomical structures, particularly cervical vertebrae, were not clearly visible on lateral cephalometric radiographs and/or with vertebral fusion or any other malformation were excluded from the study. 

The sample size for this study was calculated for 80% power and 5% significance level, considering an effect size of 0.2 on the diagnosis of third molar agenesis, as a function of dental age. According to the calculation, the required minimum sample size was 273 subjects (118 males and 155 females), while adjusting for sex. Chronologically, the sample was divided into three groups: Group 1, aged between 11.0 and 11.11 years; Group 2, aged between 12.0 and 12.11; Group 3, between 13.0 and 13.11 years. Regarding dental age, the cohort was categorized into two working groups: Group I, without full eruption of the four second molars; Group II, individuals with eruption of the four second molars. Full eruption of the four second molars was considered to have occurred when these teeth reached the occlusal plane, i.e., when radiographically the cusps of the second molars contacted the plane passing through the cusp of the first premolar and the cusps of the first molar.^10^

The stages of vertebral maturation (skeletal age) were visualized using two-dimensional lateral cephalometric radiographs and categorized according to Baccetti et al.^11^ ^,^ ^12^ for the second (C2), third (C3), and fourth (C4) cervical vertebrae. Six maturational stages of these three cervical vertebrae were identified based on the morphology of the vertebral bodies. These were defined by, firstly, examining the inferior border of the vertebral bodies to determine whether they were flat or concave, and subsequently evaluating the shape of C3 and C4: the shape of vertebral bodies transform in a characteristic sequence, developing from trapezoidal to rectangular horizontal, square, and to rectangular vertical. Typically, cervical stages CS1 and CS2 are considered prepubertal; CS3 and CS4, circumpubertal; and CS5 and CS6, postpubertal.^11^ ^,^ ^12^

The individuals in the sample were assigned to one of the six stages of maturation, i.e. CS1, CS2, CS3, CS4, CS5 and CS6, respectively. Subsequently, these six stages of cervical maturation were divided into two groups (Group A and Group B) according to the peak growth, which was considered to be between stages CS3 and CS4.^11^ ^,^ ^12^

The diagnosis of third molar agenesis was performed by using the initial first panoramic radiograph. Subsequently, the diagnosis of agenesis of third molars was confirmed or otherwise by analyzing the second panoramic radiograph obtained after 14 years of age.^14^ Correlations between the accuracy of the diagnosis and chronological, dental and or skeletal ages were performed. In this study, the third molar was considered congenitally missing when there was no evidence of radio-transparency associated with the formation of the osseous crypt^14^ on panoramic radiography or there was no evidence that the tooth had been extracted.^13^ ^,^ ^15^

## ASSESSMENT OF METHOD ERROR

An expert on CVM analysis arranged a training session for the examiner.^13^ Subsequently, a calibration test was undertaken. Thirty-six randomly selected cephalometric radiographs (not included in the present study) were re-analysed after a two-week interval, by the examiner and the expert. A kappa test revealed almost perfect inter-examiner agreement (k=0.866). A kappa agreement test was also used to determine the reproducibility of the examiner’s assessments, resulting a value of k=0.830. Examiner error was re-determined after a four-week interval for 15% randomly selected cases from the study group for all the analyzed parameters (agenesis of third molars, dental age and skeletal age). The results revealed 100% agreement between the two trials.

## STATISTICAL ANALYSIS

Statistical analysis was performed using the software IBM SPSS^®^ Statistics v. 24 (Armonk, NY, USA). To test the hypotheses initially formulated, the chi-square test of independence was used to evaluate the association between the examined parameters and third molar agenesis. A significance level of 5% was established (*p* < 0.05).

## RESULTS

Out of the 2,960 individuals screened, only 282 (9.5%) fulfilled the inclusion criteria (122 males and 160 females). The number of subjects in each group according to their chronological age was as follows (Table 1): Group 1, 81 individuals aged between 11.0 and 11.11 years (28.7%); Group 2, 106 individuals aged between 12.0 and 12.11 (37.6%); Group 3, 95 individuals aged between 13.0 and 13.11 years (33.7%). Moreover, according to dental age, the number of individuals in each group was: Group I, 141 individuals without full eruption of the four second molars (50%); Group II, 141 individuals with eruption of the four second molars (50%). Only 28 individuals (9.9% of the whole sample), with an equal number of males and females (14 each), showed a change in the diagnosis of third molar agenesis between the first and second radiographic observations. Neither sex nor age were significantly associated with a change in diagnosis (sex, *p*=0.448; age, *p*=0.175). Moreover, the distribution of the individuals in the six stages of maturation, CS1, CS2, CS3, CS4, CS5 and CS6, respectively, was as follows: 57 (20.2%), 43 (15.2%), 52 (18.4%), 49 (17.4%), 53 (18.8%) and 28 (9.9%).Table 2 illustrates that, for half the cohort, all four second molars were unerupted before the age of 14 years (Group I). Table 2 also shows that the proportion of cases in Group I where the diagnosis of third molar agenesis changed was significantly greater than in the group where the second molars had erupted before fourteen years of age (Group II). This confirms a significant association between the dental age of second molars and a change in diagnosis (*p*<0.001).

**Table 1:** Number (n) and percentage (%) of cases that had the same or different diagnosis according to sex (*p*=0.448) and age group (*p*=0.175).

	Female	Male	Total	Group 1	Group2	Group 3
	n (%)	n (%)	n (%)	11.0-11.11 (years)	12.0-12.11 (years)	13.0-13.11 (years)
Same diagnosis	146 (91.3)	108 (88.5)	254 (90.1)	71 (87.7)	93 (87.7)	90 (94.7)
Different diagnosis	14 (8.8)	14 (11.5)	28 (9.9)	10 (12.3)	13 (12.3)	5 (5.3)
Total	160	122	282	81	106	95

**Table 2:** Number (n) and percentage (%) of cases that had the same or different diagnosis according to second molar eruption (*p*<0.001).

Group	Same diagnosis n (%)	Different diagnosis n (%)	Total n
Group I: No eruption of the four second molars	115 (81.6)	26 (18.4)	141
Group II: With eruption of the four second molars	139 (98.6)	2 (1.4)	141
Total	254 (90.1)	28 (9.9)	282

The data show that the number of altered diagnoses decreased with increasing stage of CVM up to the last stage (CS6) at the end of the growth peak, where 100% of the cases maintained the same diagnosis after 14 years of age. To make this outcome less confusing and more easily applied clinically, the data were categorized into two groups: Group A, including pre-peak growth data from individuals who had not reached their peak growth (C1, C2 and C3); and Group B, comprising post-peak growth data (C4, C5 and C6) (Table 4). In Group A, 22 individuals (14.5%) out of 152 had their diagnosis of third molar agenesis changed after the age of 14 years (Table 3). By contrast, only 6 (4.6%) out of 130 individuals in Group B had their diagnosis of third molar agenesis rejected. A significant disparity between the two groups concerning diagnostic alterations of third molar agenesis was found (*p*=0.006).

**Table 3:** Number (n) and percentage (%) of cases that had the same or different diagnosis according to the six skeletal stages of mineralization.

	Same diagnosis n (%)	Different diagnosis n (%)	Total
CS1	46 (80.7)	11 (19.3)	57
CS2	37 (86.0)	6 (14.0)	43
CS3	47 (90.4)	5 (9.6)	52
CS4	45 (91.8)	4 (8.2)	49
CS5	51 (96.2)	2 (3.8)	53
CS6	28 (100.0)	0 (0.0)	28
Total	254 (90.1)	28 (9.9)	282

**Table 4:** Number (n) and percentage (%) of cases that had the same or different diagnosis before and after peak of growth (p=0.006).

Groups	Same diagnosis n (%)	Different diagnosis n (%)	Total n
Group A: Before peak growth (CS1-CS3)	130 (85.5)	22 (14.5)	152
Group B: After peak growth (CS4-CS6)	124 (95.4)	6 (4.6)	130
Total	254 (90.1)	28 (9.9)	282

## DISCUSSION

This study was a retrospective longitudinal radiographic study undertaken to investigate the correlation between chronological, dental and skeletal ages, and the early diagnosis of presence/absence of third molars in a sample of Portuguese orthodontic patients. Despite the considerable number of studies on agenesis and the development of third molars, few have evaluated the criteria used for early diagnosis of third molar agenesis. 

In the present study, only 28 individuals (9.9% of the sample, with equal numbers of both sexes) showed a change in the diagnosis of third molar agenesis between the first and second radiographs. These results are close to the 6.1% found by Rocha.^18^ A chi-squared test did not reveal a significant association between gender and diagnostic change. These findings lead to the conclusion that sex has no influence on the early diagnosis of third molar agenesis, which is in agreement with the findings of Bolaños et al.^14^ Similarly, there was no significant association between chronological age of the subjects and diagnostic change in the three age groups. The present results are in agreement with those of Rocha,^18^ who also did not find a significantly higher prevalence of diagnostic alterations in younger individuals. The present results agree with those of Garn and Lewis,^16^ who considered it unfeasible to make a reliable diagnosis of third molar agenesis before the age of 14 years. Considering the present results together with the previous studies of Bolaños et al.^14^ and Rocha,^18^ it is clear that between 11.0 and 13.11 years of age it is not possible to establish a chronological age criterion for the early diagnosis of third molar agenesis. 

Regarding dental age, in individuals whose four second molars had not yet fully erupted, there was a relatively high proportion of changed diagnoses (18.4%). By contrast, in the group in which the four second molars had already erupted, there were diagnostic changes in only two individuals (1.4%). Similar findings were reported by Rocha,^18^ who observed significant diagnostic changes only in individuals whose four second molars had not erupted, concluding that eruption of the second molars was a fundamental criterion in the diagnosis of third molar agenesis. In the present sample, there was a significant association between the adopted dental age criterion (eruption of the four-second molars) and a change in diagnosis, i.e., if the four second molars had erupted prior to the first radiograph, there was a significantly lower chance of diagnostic error. Therefore, based on the statistical evaluation, it was possible to state that, for the present cohort, the dental age determined by second-molar eruption could be used as a criterion for the early diagnosis of third molar agenesis. 

Finally, skeletal age was the last criterion evaluated in the present study. The lack of similar literature on the role of skeletal age in the diagnosis of third-molar agenesis means that there is no basis for a critical comparison of the present findings. The first evaluation of skeletal age according to the six stages of maturation^11^ ^,^ ^12^ is shown in Table 3. The number of individuals who showed diagnostic changes in CVM stages CS1, CS2, CS3, CS4, CS5 and CS6 was 11 (19.3%), 6 (14.0%), 5 (9.6%), 4 (8.2%), 2 (3.8%) and 0 (0.0%), respectively. There is thus a clear decreasing trend in the proportion of change in diagnosis with increasing level of skeletal maturation, leading to a fully consistent diagnosis of third molar agenesis between first and second radiographs when growth subsides (stage CS6). Therefore, for the present sample, the diagnosis of third molar agenesis undertaken at stage CS6 is considered reliable; an individual who has completed growth (CS6) has a higher probability of having a correct initial diagnosis than a growing individual. These observations suggest that skeletal age has an influence on the diagnosis of third molar agenesis. To extrapolate the previous observations and attempt to determine a stage earlier than CS6 as a diagnostic criterion of third molar agenesis, the cervical maturation stages were grouped. Table 4 allows a quantification of the individuals who showed a change in diagnosis for the two cervical maturation groups. For the group of individuals who had not yet reached the growth peak (Group A), the diagnosis was altered in 22 cases (14.5%), while in the group that had passed peak growth (Group B), the diagnosis was changed in only six cases (4.6%). The results demonstrate a significant association between diagnostic change and skeletal age determined by peak growth. Individuals in Group A (prepubertal stage) had a significantly higher proportion of diagnostic alterations than individuals in Group B (post pubertal stage). Considering the present results, it can be stated that skeletal age -namely postpubertal peak growth- was a criterion for early diagnosis of third molar agenesis in the present cohort. 

The outcome of the present study is that it was not possible to make a reliable diagnosis of third molar agenesis in individuals between 11 and 13 years of age (first null hypothesis accepted). However, the dental age, defined by the eruption of the four second molars, was found to be a valid diagnostic criterion of third molars agenesis (second null hypothesis rejected). Furthermore, skeletal age, determined by the maturation of cervical vertebrae, could also be used as a diagnostic criterion for third molar agenesis (third null hypothesis rejected). Early diagnosis of third molar agenesis might aid orthodontists and oral surgeons in a better case management, as the third molar persistence in specific situations could pose problems in adult life.^22^ However, further research work carried out under previously dimensionally defined subgroups and among different populations is required to verify and validate these findings; and to assess the predictive value of other contributing factors, such as skeletal relationships and crowding, on the chances of successful space closure.

Throughout this work there were some limitations. Firstly, related to the application of inclusion criteria such as the requirement for panoramic radiography and lateral cephalometric radiography to have been performed on the same day, and an age cut-off of less than 14 years. On the other hand, the absence of panoramic radiography after 14 years of age also limited the sample size. Another limitation for the present investigation was the sparsity of published studies on criteria that could facilitate the early diagnosis of third molar agenesis for clinicians and researchers. Thus, it is essential to carry out further prospective studies that investigate the role of biological age in the diagnosis of third molar agenesis. Within this context, the present findings could be used for power calculation in similar multicenter studies to validate the results.

## CONCLUSIONS

» Chronological age between 11.0 and 13.11 years is not a criterion for early diagnosis of third molar agenesis.
» Dental age, defined by the eruption of the four second molars, can be used as a criterion for early diagnosis of agenesis of third molars.
» Skeletal age, determined by peak growth, is also a criterion for early diagnosis of third molar agenesis.
